# Outcome of Husband-to-Wife Kidney Transplantation With Mutual Children: Single Center Experience Using T Cell-Depleting Induction and Review of the Literature

**DOI:** 10.3389/fmed.2021.724851

**Published:** 2021-08-02

**Authors:** Lisa Senn, Caroline Wehmeier, Gideon Hönger, Irene Geiger, Patrizia Amico, Patricia Hirt-Minkowski, Jürg Steiger, Michael Dickenmann, Stefan Schaub

**Affiliations:** ^1^Clinic for Transplantation Immunology and Nephrology, University Hospital Basel, Basel, Switzerland; ^2^HLA-Diagnostics and Immungenetics, Department of Laboratory Medicine, University Hospital Basel, Basel, Switzerland; ^3^Transplantation Immunology, Department of Biomedicine, University of Basel, Basel, Switzerland

**Keywords:** husband-to-wife transplantation, kidney transplantation, allograft rejection, infection, pregnancy-induced sensitization

## Abstract

Few data on husband-to-wife transplantations with mutual children (H2W) exist in the current era. We investigated the outcome of H2W transplantations (*n* = 25) treated with T cell-depleting induction compared to women with prior pregnancies also receiving their first HLA-mismatched kidney transplant, but from a different donor source: (i) other living donor (*n* = 52) and (ii) deceased donor (*n* = 120). Seventy-four percent of the women had ≥2 pregnancies; median follow-up time was 5 years. Death-censored allograft survival was significantly lower in the H2W group compared to the other two groups (*p* = 0.03). Three of four graft losses in the H2W group were due to rejection. 5-year patient survival in the H2W group was high and similar compared to the other living donor group (100 vs. 98%; *p* = 0.28). The incidence of (sub)clinical antibody-mediated rejection was higher in the H2W group (36 vs. 20 vs. 18%) (*p* = 0.10). The frequency of infections was similar among the three groups. No immunological parameter was predictive for rejection or graft loss in H2W transplantations. In conclusion, H2W transplantation is a valuable option, but associated with a higher risk for allograft loss due to rejection despite T cell-depleting induction. Further research is required for better risk prediction on an individual patient level.

## Introduction

Pregnancy is an important reason for HLA-directed sensitization. Using sensitive single antigen bead assays on the Luminex platform, child-specific HLA-directed antibodies are detected immediately after delivery in about 20–30% of women after one pregnancy and almost 50% after three or more pregnancies (1). This suggests that repeated exposures to the same HLA molecules increases the likelihood of a detectable humoral immune response (2, 3). Furthermore, it is well-known that pregnancy-induced HLA antibodies can diminish over time, while alloreactive T- and B cells still persist (4).

Many women evaluated for kidney transplantation had prior pregnancies more than 10–20 years ago (5, 6). As sera dating back to the immediate time after delivery are very rarely available, sensitization cannot be excluded, even if no HLA antibodies are detectable in current sera. This is a major diagnostic challenge in husband-to-wife transplantations with mutual children (H2W), because there will be a 20–50% chance of prior husband-specific sensitization depending on the number of pregnancies. Indeed, several case reports/series demonstrated that severe early rejection can occur in H2W transplantations despite the absence of detectable HLA antibodies prior to transplantation (7–10).

Although H2W transplantations carry an unpredictable risk of rejection, some women do not have other options as living donors. In addition, the benefit of a preemptive/timely H2W transplantation compared to a yearlong dialysis period and subsequent deceased donor transplantation has to be considered. At our center, we regard H2W transplantations as immunological risk and treat these women with T cell-depleting induction. This therapy has been shown to reduce the incidence and severity of rejection in patients with donor-specific HLA antibodies (DSA) (11). So far, outcomes of H2W transplantations receiving T cell-depleting induction have not been described.

The aim of this study was to investigate pertinent outcomes of H2W transplantations treated with T cell-depleting induction in comparison to other options for women with prior pregnancies receiving their first HLA-mismatched kidney transplant (i.e., other living donor or deceased donor transplantation).

## Patients and Methods

### Patient Population

The ethics committee of Northwestern and Central Switzerland approved this retrospective study (www.eknz.ch; project ID 2021-00584). The pregnancy history is documented in all women evaluated for kidney transplantation. In case of spousal donors, we carefully evaluated during the living donor evaluation process, if the spouse was indeed the biological father of the children (i.e., separate interviews of the couple by a physician and a dedicated psychologist).

For this study, we assessed all kidney transplantations in women performed at the University Hospital Basel from January 1st, 2005 until January 31st, 2020 for eligibility (*n* = 368). One-hundred and seventy-one of 368 transplantations (46%) were excluded for the following reasons: no previous pregnancies (*n* = 85), previous transplantation(s) (*n* = 56), induction protocol violation (*n* = 19; detailed in the immunosuppression section), HLA-identical living donor transplantation (*n* = 8), child-to-mother transplantation (*n* = 2), and unknown pregnancy status (*n* = 1). The remaining 197 women all had their first HLA-mismatched kidney transplantation and previous pregnancies. According to the kidney donor source and the detailed pregnancy history the transplantations were divided into three groups: (i) H2W (*n* = 25), (ii) other living donor (*n* = 52), (iii) deceased donor (*n* = 120).

### Living and Deceased Donor Selection Process

HLA antibody analysis was performed by single antigen beads on the Luminex platform using a cutoff of 500 MFI, and DSA were determined by a virtual cross-match approach as previously reported (5, 12).

All willing and medically eligible living donors are usually evaluated regarding histocompatibility. Priority is given to donors without DSA constellation. Husbands having mutual children with the recipient were accepted as donors, if no DSA constellation was present. If DSA were present, transplantation was pursued after discussion with the couple regarding other options, and if considered as immunologically feasible (negative T- and B-cell CDC-cross-matches, and usually no more than three DSA at ≤2 loci and cumulative MFI<10000). Other living donors with DSA were accepted using the same criteria.

For deceased donor selection, priority is given to DSA negative donors according to the algorithm of the national donor allocation program (13). DSA were accepted in patients with high cPRA, if regarded as immunologically feasible (negative T- and B-cell CDC-crossmatches) (12).

### Immunosuppression

H2W transplantations were considered as immunological risk and received an induction therapy consisting of a polyclonal anti T cell globulin (ATG; Gravalon 9 mg/kg bw prior to reperfusion of the allograft and 3 mg/kg bw on day 1–4 or Thymoglobulin 4 days 1.5 mg/kg bw). In case of circulating DSA, intravenous immunoglobulins (IvIg) were additionally given (5 days 0.4 g/kg bw). Maintenance immunosuppression consisted of tacrolimus (Tac), mycophenolate (MPA) and prednisone. Target tacrolimus trough levels were 10–12 ng/ml for the first month, 8–10 ng/ml for months two to three, 6–8 ng/ml for months four to six, and 4–8 ng/ml thereafter. Steroids were tapered to 0.1 mg/kg body weight by month three post-transplant.

For all other transplantations, the induction therapy was selected based on the presence/absence of DSA. Patients without DSA received an induction therapy with basiliximab (20 mg on day 0 and 4) and triple therapy with Tac-MPA-P or a steroid-free regimen consisting of Tac-MPA and a mTOR-inhibitor. In case of a rejection-free clinical course, immunosuppression was modified and reduced within the first 6 months to establish a dual Tac-MPA therapy on the long-term. Target trough levels of tacrolimus were identical to the levels described above. Patients with DSA received an induction therapy with ATG and IvIg and maintenance immunosuppression consisting of Tac-MPA-P. Target trough levels of tacrolimus were identical to the levels described above. Steroids were tapered to 0.1 mg/kg body weight by month three post-transplant and maintained at this level.

All ABO-blood group incompatible (ABOi) transplant recipients received a single dose of rituximab 4 weeks prior to transplantation and immunadsorption depending on the anti-blood group titers.

As mentioned above, 19 transplantations were excluded from the study, because the used induction deviated from the protocol. Most of these violations occurred in deceased donor transplantations due to retrospective DSA status corrections after extending the donor HLA typing (i.e., additional loci or high resolution typing) (14). Only one patient in the H2W group was excluded. This patient received an ABOi transplant without DSA and the induction was restricted to rituximab without additional ATG due to her frailty.

### Medication as Infection Prophylaxis

All patients received prophylaxis with trimethoprim/sulfamethoxazole (160/800 mg three times per week) against pneumocystis jirovecii infection for 6 months. The CMV prevention strategy at our center has been described previously (15). Briefly, high-risk patients (D+/R–) received prophylaxis with oral valganciclovir (Valcyte, Roche) 450 mg twice daily adjusted for renal function. Intermediate-risk patients (R+) received prophylaxis with valganciclovir, if they had an induction therapy with ATG or were ABO-incompatible. All other intermediate-risk patients were managed by regular monitoring and deferred therapy. Low-risk patients (D–/R–) received no prophylaxis and had no regular screening. Prophylaxis was given for a minimum of 3 months and prolonged, if immunosuppression was still considered as high (e.g., recent rejection therapy).

### Assessment and Treatment of Allograft Rejection

Patients were monitored by surveillance biopsies at 3 and 6 months post-transplant. Clinically indicated allograft biopsies were performed when serum creatinine increased by >20% from baseline. Findings were graded according to the Banff 2015 classification (16). Mixed rejection were grouped to antibody-mediated rejection (ABMR). Clinical and subclinical rejection episodes were treated according to the phenotype and severity. Clinical T cell-mediated rejection (TCMR) were mainly treated with i.v. steroid pulses (3-5^*^500 mg methylprednisolone) and a steroid taper. Subclinical TCMR were mainly treated with p.o. steroids (3^*^200 mg prednisone) and a steroid taper. Patients with clinical ABMR and mixed rejection episodes received ATG and in some cases in addition IvIg. Subclinical ABMR or mixed rejection were treated with i.v. or p.o. steroid pulses and a steroid taper. Borderline changes, which are by far the most frequent rejection phenotype in the current era of immunosuppression, were regarded and treated as TCMR (17).

### Statistical Analysis

We used JMP software (SAS Institute Inc., Cary, NC) for statistical analysis. Categorical data are presented as counts and/or percentages and were analyzed by chi-square test or Fisher's exact test as appropriate. Continuous data are shown as median and interquartile ranges [IQR] and compared by Wilcoxon rank sum tests. For all tests, a (two-tailed) *p* < 0.05 was considered to indicate statistical significance. Time-to-event analyses were performed by the Kaplan-Meier method and compared by the log-rank test. Multivariate Cox regression analysis was used to investigate independent predictors for rejection and graft failure in H2W transplantations.

## Results

### Baseline Characteristics

Baseline characteristics of the three groups are summarized in Table 1. We observed significant differences regarding several parameters. Recipients in the deceased donor and H2W groups were older than in the other living donor group (59 vs. 56 vs. 49 years; *p* < 0.0001). In addition, women in the H2W and deceased donor groups had more often ≥2 pregnancies compared to the other living donor group. As expected, the latter group had less HLA mismatches than the other groups due to related in-family donors (i.e., parents, siblings). We observed no statistically significant differences regarding the frequency of DSA as well as their characteristics. CMV constellations were equally distributed among the three groups, but prophylaxis with valganciclovir was given more often in the H2W group due to universal use of ATG induction. Maintenance immunosuppression with Tac and MPA was used in 194/197 patients (98%).

**Table 1:** Baseline characteristics.

**Parameter**	**Husband-to-wife**	**Other living donor**	**Deceased donor**	***p***
	**(*n* = 25)**	**(*n* = 52)**	**(*n* = 120)**	
Recipient age	56 (47–64)	49 (39–57)	59 (53–65)	<0.0001
**Renal disease**				
ADPKD	8 (32%)	16 (31%)	28 (23%)	0.48
Diabetic nephropathy	1 (4%)	7 (13%)	13 (11%)	
Glomerulonephritis	6 (24%)	15 (29%)	33 (28%)	
Interstitial nephropathy	-	3 (6%)	8 (7%)	
Vascular nephropathy	2 (8%)	3 (6%)	13 (11%)	
Other nephropathies	4 (16%)	6 (12%)	8 (6%)	
Unknown nephropathy	4 (16%)	2 (3%)	17 (14%)	
**Renal replacement therapy**				
Preemptive transplantation	12 (48%)	22 (42%)	5 (4%)	<0.0001
Dialysis vintage time [years]	0.8 (0.2–1.8)	0.7 (0.2–1.4)	2.6 (1.6–4.3)	<0.0001
Donor age	60 (51–66)	52 (46–59)	53 (25–66)	0.15
**Number of pregnancies**				
One	4 (16%)	23 (44%)	23 (19%)	0.004
Two	13 (52%)	19 (37%)	50 (42%)	
≥3	8 (32%)	10 (19%)	47 (39%)	
Blood transfusions (yes/no/unknown)	29%/54%/17%	24%/69%/8%	40%/42%/18%	0.03
cPRA (A/B/DR/DQ) [%]	1 (0–48)	20 (0–42)	23 (0–78)	0.03
ABO incompatible	2 (8%)	10 (19%)	n.a.	n.a.
DSA present	9 (36%)	8 (15%)	28 (23%)	0.13
DSA characteristics	*n* = 9	*n* = 8	*n* = 28	
Number	2 (1–3)	2 (1, 2)	1 (1, 2)	0.48
Class (I/II/I+II)	3/3/3	1/3/4	10/13/5	0.40
Cumulative MFI	1,571 (889–7,023)	1,533 (1,014–4,269)	1,425 (597–4,646)	0.80
HLA mismatches (A/B/DR/DQ)	6 (5–7)	4 (3–6)	5 (4–6)	0.004
**CMV risk constellation**				
High risk (D+/R–)	4 (16%)	7 (14%)	15 (13%)	0.71
Intermediate risk (R+)	14 (56%)	37 (71%)	80 (67%)	
Low risk (D–/R–)	7 (28%)	8 (15%)	25 (21%)	
Prophylaxis with valganciclovir	18 (72%)	21 (40%)	41 (34%)	0.002
**Induction therapy**				
ATG ± IvIg	25 (100%)	8 (15%)	28 (23%)	<0.0001
Basiliximab	-	44 (85%)	92 (77%)	
**Maintenance immunosuppression**				
Tac-MPA-P	25 (100%)	40 (77%)	114 (95%)	0.0007
Tac-MPA-mTOR	-	11 (21%)	4 (3%)	
Other^*^	-	1 (2%)	2 (2%)	

*ADPKD, autosomal polycystic kidney disease; DSA, donor-specific HLA-antibodies; ATG, anti T-cell globulin; IvIg, intravenous immunoglobulins; Tac, tacrolimus; MPA, mycophenolic acid; mTOR, mTOR inhibitors; P, prednisone; n.a., not applicable*.

* *No Tac-based immunosuppression*.

### Patient and Graft Survival

**Figure 1:**
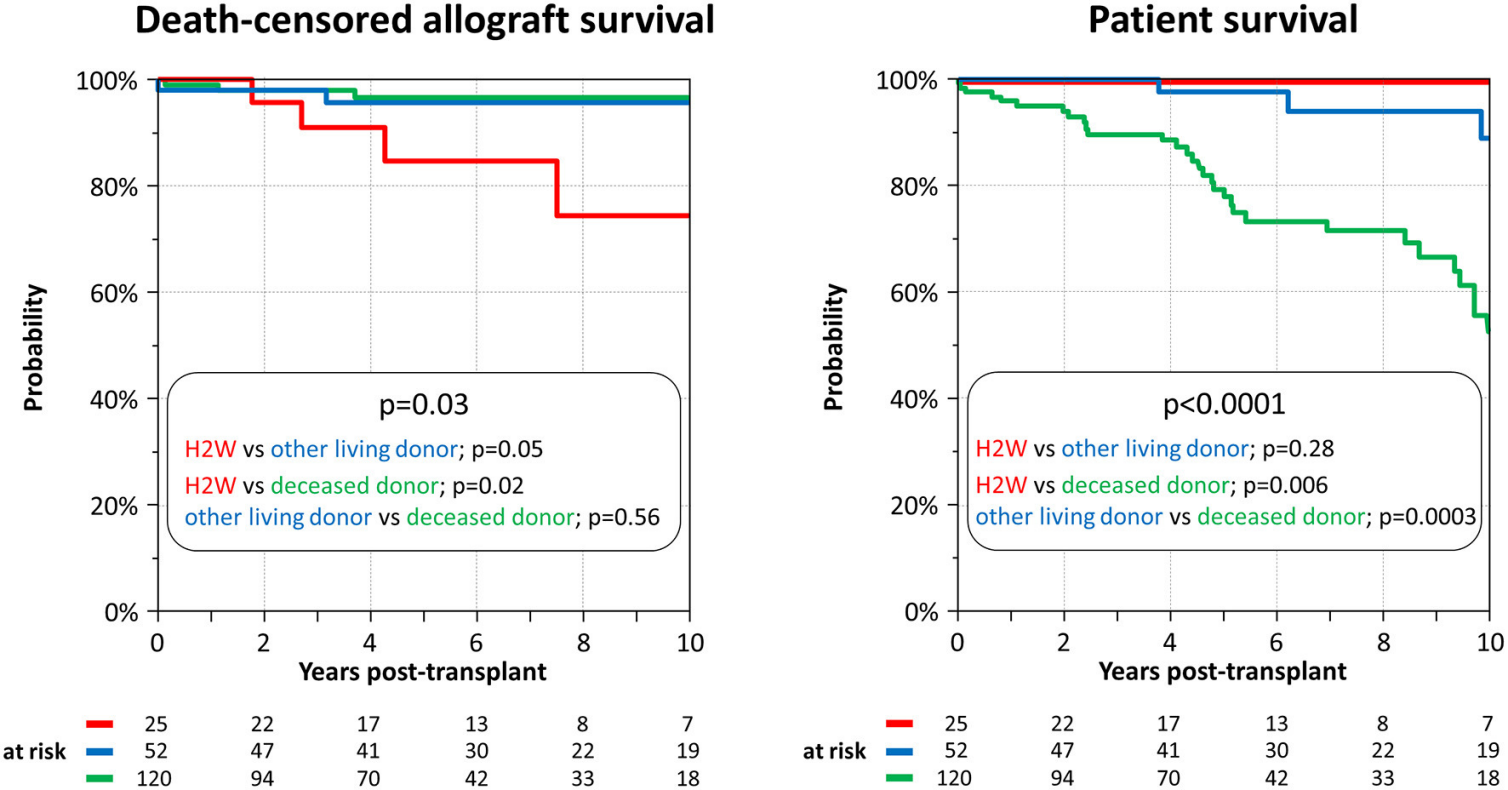
Death-censored allograft and patient survival.

After a median follow-up time of 5.1 years (IQR 2.9–9.0), 11/197 allograft (5.6%) failed: four in the H2W group, two in the other living donor group, and five in the deceased donor group. Rejection accounted for 6/11 graft failures (55%), three of these occurred in the H2W group. One of these three patients had DSA (Cw4 and DR15 with cumulative MFI of 5759), the other two patients had no DSA. Details on the reasons for graft loss are summarized in Table 2. Death-censored allograft survival was significantly lower in the H2W group compared to the other two groups (*p* = 0.03). In detail, 1-year survival was still similar among the three groups (100 vs. 98 vs. 99%), but lower in the H2W group at five (85 vs. 96 vs. 97%) and 10 years (74 vs. 96 vs. 97%) (Figure 1).

**Table 2:** Major outcomes.

**Parameter**	**Husband-to-wife**	**Other living donor**	**Deceased donor**	***p***
	**(*n* = 25)**	**(*n* = 52)**	**(*n* = 120)**	
**Reason for graft failure**	***n*** **=** **4**	***n*** **=** **2**	***n*** **=** **5**	
Rejection	3	1	2	n.a.
Recurrent GN	1	–	–	
Vascular/surgical	–	1	–	
Other	–	–	3	
**Cause of death**	***n*** **=** **0**	***n*** **=** **4**	***n*** **=** **33**	
Cardiovascular	–	–	6	n.a.
Malignancy	–	1	5	
Infection	–	1	11	
Other	–	1	4	
Unknown	–	1	7	
**Estimated GFR**				
At 1 year	54 (39–68)	58 (47–70)	54 (38–70)	0.24
At 3 years	50 (37–62)	61 (46–75)	54 (32–72)	0.16
At 5 years	58 (38–70)	55 (43–75)	51 (33–83)	0.60
**Urine protein/creatinine ratio [mg/mmol]**				
At 1 year	10 (8–19)	13 (8–18)	13 (8–24)	0.50
At 3 years	11 (6–22)	11 (8–15)	13 (9–23)	0.20
At 5 years	16 (8–20)	9 (5–23)	13 (9–23)	0.25
**Post-transplant DSA screening**	***n*** **=** **18**	***n*** **=** **34**	***n*** **=** **73**	
No *de novo* DSA	10 (56%)	26 (76%)	44 (60%)	0.27
*De novo* DSA	1 (6%)	2 (6%)	10 (14%)	
Persisting pre-transplant DSA	3 (16%)	1 (3%)	11 (15%)	
Disappearing pre-transplant DSA	4 (22%)	5 (15%)	8 (11%)	

Thirty-seven of 197 patients (19%) died. None in the H2W group, 4 in the other living donor group and 33 in the deceased donor group. Leading causes of death were infections (*n* = 12), cardiovascular diseases (*n* = 6), and malignancies (*n* = 6) (Table 2). Patient survival was significantly lower in the deceased donor group compared to the other two groups (*p* < 0.0001) (Figure 1). Notable, patient survival was similar in the H2W and the other living donor group (*p* = 0.28).

The median estimated glomerular filtration rate (eGFR) and proteinuria of functioning allograft were similar among the three groups at one, three and 5 years post-transplant (Table 2).

### Allograft Rejection

Overall, the 197 patients had 81 clinical biopsies within the first year as well as 259 surveillance biopsies at 3 and 6 months, respectively. Only 37/197 patients (19%) had no allograft biopsy at all within the first year post-transplant. The frequency of clinical and surveillance biopsies was not different among the three groups (*p* = 0.20 and *p* = 0.38). Clinical biopsies beyond the first year post-transplant were obtained in 34/197 patients (17%), with similar frequency among the three groups (*p* = 0.64).

**Figure 2:**
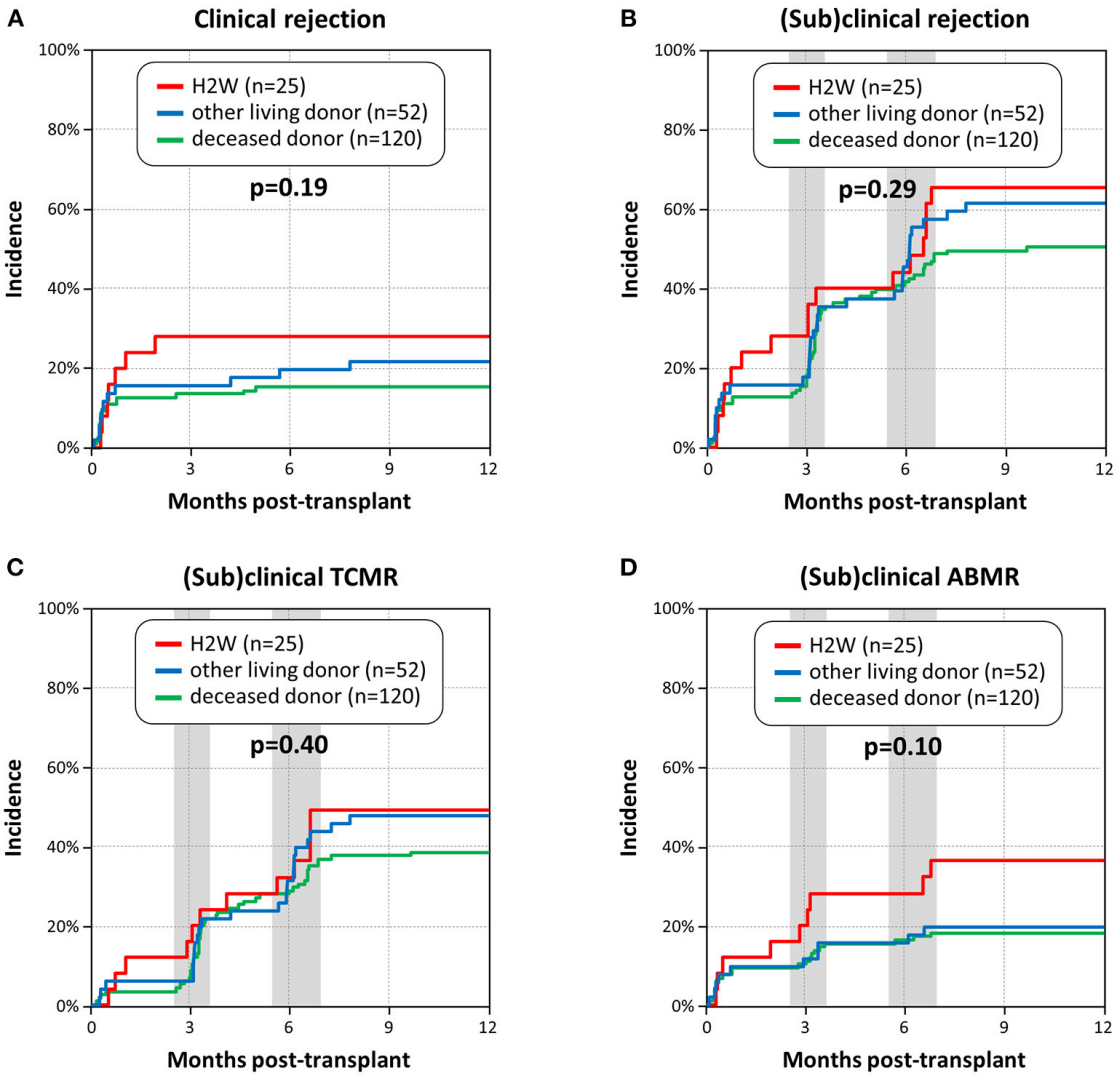
Incidence of rejection within the first year post-transplant. **(A)** Incidence of clinical rejection. **(B)** Incidence of (sub)clinical rejection. **(C)** Incidence of (sub)clinical T cell-mediated rejection (TCMR). **(D)** Incidence of (sub)clinical antibody-mediated rejection (ABMR), including mixed rejection. The gray background areas represent the two time frames, in which surveillance biopsies at 3 and 6 months were performed.

The 1-year incidence of clinical rejection was statistically not different among the three groups (*p* = 0.19), but numerically highest in the H2W group (28 vs. 22 vs. 15%). We observed similar incidences of (sub)clinical rejection and (sub)clinical TCMR (*p* = 0.29 and *p* = 0.40) among the three groups. Interestingly, the incidence of (sub)clinical ABMR was numerically around twice as high in the H2W group compared to the other groups (36 vs. 20 vs. 18%), but this did not reach statistical significance (*p* = 0.10) (Figure 2).

### Infections Within the First Year

Infections occurring within the first year post-transplant are summarized in Table 3. We observed no differences regarding frequency and severity of polyomavirus BK infection (BKV). Cytomegalovirus (CMV) infections were more frequent and more severe in the deceased donor group compared to the other groups (*p* = 0.01). However, they were equally often observed in the H2W and the other living donor group.

**Table 3:** Infections occurring within the first year post-transplant.

**Parameter**	**Husband-to-wife**	**Other living donor**	**Deceased donor**	***p***
	**(*n* = 25)**	**(*n* = 52)**	**(*n* = 120)**	
**Polyomavirus BK**				
No relevant viruria (i.e., no Decoy cells)	18 (72%)	34 (65%)	86 (72%)	0.29
Viruria, but no viremia	6 (24%)	13 (25%)	15 (13%)	
Viremia	1 (4%)	4 (8%)	17 (14%)	
Definitive BK-nephropathy	–	1 (2%)	2 (2%)	
**CMV**				
No CMV viremia	21 (84%)	43 (83%)	73 (61%)	0.01
Asymptomatic CMV viremia	3 (12%)	9 (17%)	27 (22%)	
CMV syndrome	–	–	12 (10%)	
Tissue-invasive CMV disease	1 (4%)	–	8 (7%)	
**Other infections including lower UTI**				
None	8 (32%)	18 (35%)	31 (26%)	0.24
Only one	6 (24%)	19 (37%)	32 (27%)	
≥2 infections	11 (44%)	15 (29%)	57 (47%)	
**Other infections excluding lower UTI**				
None	13 (52%)	29 (56%)	66 (55%)	0.22
Only one	5 (20%)	18 (34%)	29 (24%)	
≥2 infections	7 (28%)	5 (10%)	25 (21%)	

*UTI, urinary tract infection*.

We recorded 345 infections other than BKV and CMV. The percentage of patients having no, one or ≥2 infections was not different among the three groups (*p* = 0.24). As expected, lower urinary tract infections (UTI) were the most frequent infection accounting for 54% of all cases, followed by upper airway infections (13%), gastrointestinal infections (11%), skin infections (9%), and upper UTI (i.e., pyelonephritis) with 7%. Fungal infections were rare (15/345; 4.3%). When excluding lower UTI, about 50% patients in the three groups experienced no infection within the first year post-transplant (Table 3).

### Evolution of Pre-transplant DSA and Development of *de novo* DSA

Post-transplant screening for HLA antibodies was performed in 125/197 patients (63%), including 35/45 women having pre-transplant DSA (78%) and 90/152 women without pre-transplant DSA (59%). The frequency of screening was similar among the three groups (H2W 72%, other living donor 65%, deceased donor 61%; *p* = 0.54) and was performed at a median of 3.1 years post-transplant. Of 35 patients with pre-transplant DSA, 3 developed *de novo* DSA (9%), 15 had persisting DSA (43%), and in 17 cases DSA disappeared (48%). In those 90 women without pre-transplant DSA, 10 developed *de novo* DSA (11%). The frequency of *de novo* DSA, persisting or disappearing DSA was not different among the three groups (*p* = 0.27) (Table 2).

### Predictors of Rejection and Graft Failure in H2W Transplantations

During the whole follow-up 16/25 H2W transplantations developed (sub)clinical rejection, 10/25 clinical rejection, and 4/25 allograft failed. DSA status (yes/no), number of pregnancies (1 vs. ≥2), and HLA-mismatches were not associated with any of the above mentioned events in univariate analysis and multivariate Cox-models (all *p* ≥ 0.09).

### Review of the Literature

We found 10 publications investigating H2W transplantations with prior pregnancies: 5 cohort studies, 4 detailed case reports, and one case series (Table 4).

**Table 4:** Review of the literature.

**References**	**Study type**	**H2W (*n*)**	**Number of pregnancies**	**Husband as biological father confirmed**	**Pre-transplant DSA assessed**	**With DSA**	**Induction**	**Maintenance immuno-suppression**	**Patient survival**	**Graft survival**	**Key observation**
This manuscript	Cohort study	25	1 preg: 16% 2 preg: 52% ≥3 preg: 32%	Yes	Yes (Luminex SA)	36%	ATG ± IvIg	Tac-MPA-P	1 year: 100% 5 years: 100%	1 year: 100% 5 years: 85%	Death-censored graft survival lower in H2W compared with other living or deceased donors
Kim et al. (18)	Cohort study	159	At least one (no details given)	No	Yes (method not reported; likely solid-phase assays)	32%	ATG (4%) Basiliximab (96%)	Tac-MPA-P (79%)	5 years: 98%	5 years: 96%	No difference in patient and graft survival compared with offspring-to-mother (*n* = 175) and other living unrelated donors (*n* = 56), but higher rate of clinical rejection
Ghafari et al. (19)	Cohort study	9	2–5 (mean 2.6)	No	Yes (AHG-CDC-XM)	0%	None	CyA-MPA/AZA-P	1 year: 97% 5 years:78%	1 year: 78% 5 years: 69%	More rejection and lower graft survival compared to other living unrelated donors
Pretagostini et al. (20)^*^	Cohort study	33	At least one (no details given)	No	Yes (CDC-XM)	0%	Not reported	CyA-based	Not reported	1 year: 80% 5 years: 73%	Lower 1 year graft survival compared to W2H (90%) and other unrelated living donor (100%)
Gjertson et al. (21)	Cohort study	407	1 preg: 31% ≥2 preg: 69%	No	Yes (CDC-XM)	0%	Not reported	Not reported	Not reported	1 year: 90% 5 years: 78%	No difference in graft survival according to the number of pregnancies in H2W
Terasaki et al. (22)	Cohort study	75	At least one (no details given)	No	Not reported (likely CDC-XM)	n.a.	Not reported	Not reported	Not reported	1 year: 87% 3 years: 76%	Graft survival @3 years worse compared to H2W without any pregnancies (76% vs. 87%; *p* = 0.40)
Ortiz-Arroyo et al. (10)	Case series	5	At least one (no details given)	No	Yes (AHG-CDC-XM)	0%	Basiliximab (40%)	Triple therapy	Not reported	1 year: 60%	Two out of five patients developed acute ABMR and lost the graft
Matsuo et al. (9)	Case report	1	2	Yes	Yes (FCXM/FlowPRA)	No	Basiliximab	Tac-MPA-P	Good function at 6mt		Acute ABMR starting POD 3, reversed by plasmapheresis, steroids and rituximab
Rosenberg et al. (8)	Case report	1	5	Yes	Yes (CDC-XM/FCXM)	No	Not reported	Tac-MPA-P	Good function at 2 year		Acute rejection (Banff IIA) starting POD 2, reversed by plasmapheresis
Habicht et al. (7)	Case report	1	3	Yes	Yes (CDC-XM/FCXM)	No	None	CyA-MPA-P	Good function at 1 year		Acute ABMR (only C4d positive) POD 7, reversed by steroids, ATG and immunoadsorption
Böhmig et al. (23)	Case report	1	5	Yes	Yes (CDC-XM/FCXM)	Yes^**^	None	CyA-MPA-P	Good function at 3mt		Acute mixed rejection first week, reversed by ATG and immunoadsorption

*H2W, husband-to-wife transplantation with previous pregnancies; W2H, wife-to-husband transplantation; CDC-XM, complement-dependent cytotoxicity crossmatch; FCXM, flowcytometric crossmatch; POD, post-operative day*.

* *These authors previously reported outcomes of H2W from the same population with fewer cases (24, 25)*.

** *Retrospectively detected by FCXM*.

Nine of ten reports were published between 1995 and 2008. In these studies, a Tac- or cyclosporine-based immunosuppression without induction therapy was used for most patients. All patients were considered as having no pre-transplant DSA by either cytotoxicity or flowcytometric crossmatches. The five cohort studies or case series published between 1995 and 2008 reported 1-year graft survival from 60 to 90%, which was mostly lower than in the comparison groups and lower than in our study (100%) (10, 19–22). All case reports described acute antibody-mediated or mixed rejection episodes within the first week post-transplant, which could be successfully reversed (7–9, 23).

The only more recent publication in 2020 by Kim et al. reported on 159 H2W transplantations using Tac-MPA-P immunosuppression and basiliximab induction (18). They observed a higher frequency of clinical rejection in the H2W group, but similar patient and graft survival compared to offspring-to-mother and other living unrelated donors.

## Discussion

The key observation is this study was that despite T cell-depleting induction, H2W transplantations have a higher risk for death-censored graft loss possibly mediated by a higher frequency of ABMR compared to other HLA-mismatched living or deceased donor transplantations in women with prior pregnancies. However, patient survival in H2W transplantation is excellent, and the universal use of T cell-depleting induction was not associated with a higher frequency or infections within the first year post-transplant.

Previous cohort studies published between 1995 and 2008 reported 1-year graft survival rates between 78 and 90% in H2W transplantations, which were mostly lower than in the comparison groups (19–22). Although the frequency of rejection was often not reported, we assume that most of these 10–20% early graft losses might be related to rejection, which were not prevented by the used immunosuppressive protocol. We speculate that ATG induction together with Tac-MPA-P maintenance immunosuppression contributed to the better 1-year graft survival (i.e., 100%) in our study. Support for this interpretation comes from a retrospective cohort study, which demonstrates the efficacy of ATG induction in allograft recipients with pre-existing donor-specific sensitization (11). Clearly, only a prospective randomized study in H2W transplantations comparing ATG vs. other induction (e.g., basiliximab) or no induction can provide more conclusive evidence, but it is very unlikely that such a study will ever be performed.

A recent study from South Korea reported excellent short and long-term graft survival in 159 H2W transplantations receiving basiliximab induction and Tac-MPA-P maintenance immunosuppression (18). We cannot explain these favorable results compared to reports from Europe and North America. One possibility might be that South Korea has a less heterogeneous population and hence less immunogenic HLA mismatches. Furthermore, the number of pregnancies was not reported in their study. A lower frequency of prior pregnancies will reduce the overall risk of a husband-specific sensitization, which can critically influence the outcome (1).

Our study as well as most of the referenced publications highlight that H2W transplantations are associated with a wide range of outcomes, ranging from early, severe rejection and graft loss to uneventful courses. Unfortunately, currently available parameters such as presence of DSA, number of pregnancies, and number of HLA mismatches seem not to be very predictive. Analyses on the molecular level of HLA disparities (i.e., eplet load and eplet immunogenicity) might provide better prediction, but such an evaluation would require a significantly larger number of cases (26–30). Another interesting approach could be the evaluation of memory B cell responses (31). With recent advances to detect memory B cells, it seems nowadays possible to obtain a more complete picture of the pre-transplant alloimmunization status in these women (32–35). Since the magnitude of memory B cell responses following previous HLA immunization such as pregnancies may differ, their detection might explain the higher proportion of early (sub)clinical rejection episodes in H2W transplants, as observed in our study and described before (7–10, 23).

How should we council pairs in evaluation for a H2W transplantation? If the husband is indeed the biological father of the mutual child/children, we recommend to use an appropriate immunosuppressive regimen including ATG induction and to discuss with the pair the increased risk for rejection as well as allograft failure. Alternative living donors having different HLA haplotypes than the biological father should be prioritized, especially in case of numerous pregnancies. If a broad and/or high husband-specific sensitization can be detected by single antigen bead assays and no alternative suitable living donor is available, a deceased donor transplantation or inclusion in a kidney-paired donation program avoiding all major DSA might be the best option (36). Avoidance of even all mismatched husband HLA antigens (despite lack of detectable husband-specific sensitization by the most sensitive techniques) by opting for deceased donor transplantation is probably the safest approach to prevent early severe rejection (37). However, this will often significantly reduce the pool of suitable donors. From a statistical point of view, a woman with two full-term pregnancies has a 40% risk to be sensitized against any HLA-A/B/C/DRB1 mismatches of the father (1). If a deceased donor shares only one HLA mismatch with the father, the risk of performing the transplantation in the presence of pre-existing sensitization will be much lower in most cases, but it still exists (37).

Despite a numerically higher incidence of ABMR in the H2W group compared to the other two groups, the frequency of detectable circulating DSA post-transplant was not different. Notably, post-transplant screening for DSA was not performed at the time of allograft biopsies, but mainly beyond the first year post-transplant after a median of 3.1 years. It is possible that the frequency of circulating DSA post-transplant is underestimated due to absorbance of circulating DSA in the allograft.

Patient survival in the deceased donor group was rather low, but death-censored allograft survival excellent (Figure 1). Notably, the women in this group were significantly older (median 59 years) and had longer dialysis vintage time (median 2.6 years) compared to the other groups. Therefore, they had very likely more relevant comorbidities and a poorer health condition, which were responsible for the high mortality. Patient death is the leading cause of allograft loss in elderly patients, which will consequentially lead to a high death-censored allograft survival (38).

This study has certain limitations. First, as in all previous reports, H2W transplantations represent a real-life selection, and it is difficult to define appropriate control groups. To reduce biases, we decided to include all women with prior pregnancies, who received the first HLA-mismatched kidney transplant and grouped them according to the donor source. The defined groups (H2W, other living donor, and deceased donor) essentially delineate the three possible options for women with prior pregnancies. Second, several analyses have clear statistical limitations due to a low patient number in the H2W group (*n* = 25) and/or low event rates. In particular, the absence of significant predictors of rejection and allograft failure in H2W transplantations has to be interpreted with caution. Third, review of the literature revealed that important parameters such as number of pregnancies, definition and presence of DSA, and induction therapy are incompletely reported, making comparison between studies difficult.

In conclusion, H2W transplantation with mutual children is a valuable option, but is associated with a higher risk of allograft loss due to rejection despite T cell-depleting induction. Further research is required to better predict this risk on an individual patient level.

## Data Availability Statement

The raw data supporting the conclusions of this article will be made available by the authors, without undue reservation.

## Ethics Statement

The studies involving human participants were reviewed and approved by Ethics committee of Northwestern and Central Switzerland (www.eknz.ch); project ID 2021-00584. Written informed consent for participation was not required for this study in accordance with the national legislation and the institutional requirements.

## Author Contributions

LS and SS: designed study, performed research, analyzed data, and wrote the manuscript. LS, CW, GH, PA, PH-M, and IG: collected data. CW, GH, IG, PA, PH-M, JS, and MD: critically reviewed and revised the manuscript. All authors contributed to the article and approved the submitted version.

## Conflict of Interest

The authors declare that the research was conducted in the absence of any commercial or financial relationships that could be construed as a potential conflict of interest.

## Publisher's Note

All claims expressed in this article are solely those of the authors and do not necessarily represent those of their affiliated organizations, or those of the publisher, the editors and the reviewers. Any product that may be evaluated in this article, or claim that may be made by its manufacturer, is not guaranteed or endorsed by the publisher.
